# Supplementary figures and images for: Distribution of calbindin-positive neurons across areas and layers of the marmoset cerebral cortex
Source: PLoS Comput Biol. 2024 Sep 23;20(9):e1012428. doi: 10.1371/journal.pcbi.1012428 (PMC11495585; doi:10.1371/journal.pcbi.1012428)

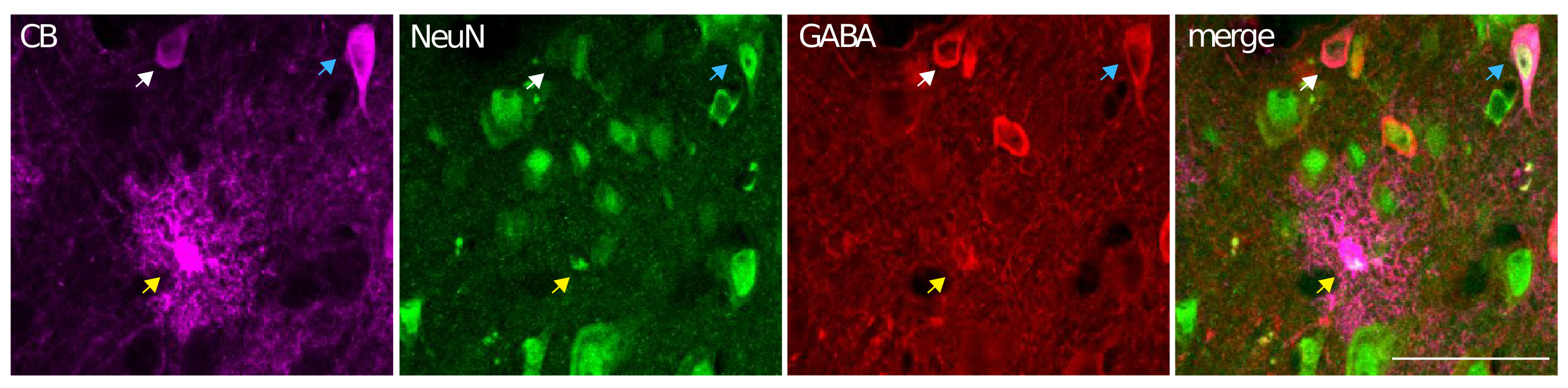

Supplement: S1 Fig — Confocal images for colocalization of calbindin-positive (CB+) neurons with neuronal marker (NeuN) and marker for inhibitory neurons (GABA) taken from primary motor cortex (A4ab). Color-coded arrows point to several CB+ neurons, including an example of a neurogliaform neuron (yellow arrow). Scale bar: 50 μm. Primary antibodies used to create the images are: Calbindin D28K, (CB, 1:100 from Thermo Fisher, Cat# PA1-931, RRID: AB_2068509), Neuronal marker (NeuN, 1:700 from Millipore, Cat# MAB377, RRID: AB_2298772), gamma aminobutyric acid (GABA, 1:500 from Sigma-Aldrich, Cat# A2052 RRID: AB_477652). For fluorescence staining, sections were incubated in blocking solution (0.3% Triton-X100 and 10% horse serum in 0.1 phosphate buffer solution) for 1 h at room temperature, followed by 46–48 h incubation in primary antibodies. The secondary antibodies [1:600, Alexa Fluor 488 (ab150109), Alexa Fluor 594 (ab150064) and Alexa Fluor 647 (ab150111)] were applied for 60 min at room temperature. (TIF) [file pcbi.1012428.s001.tif]

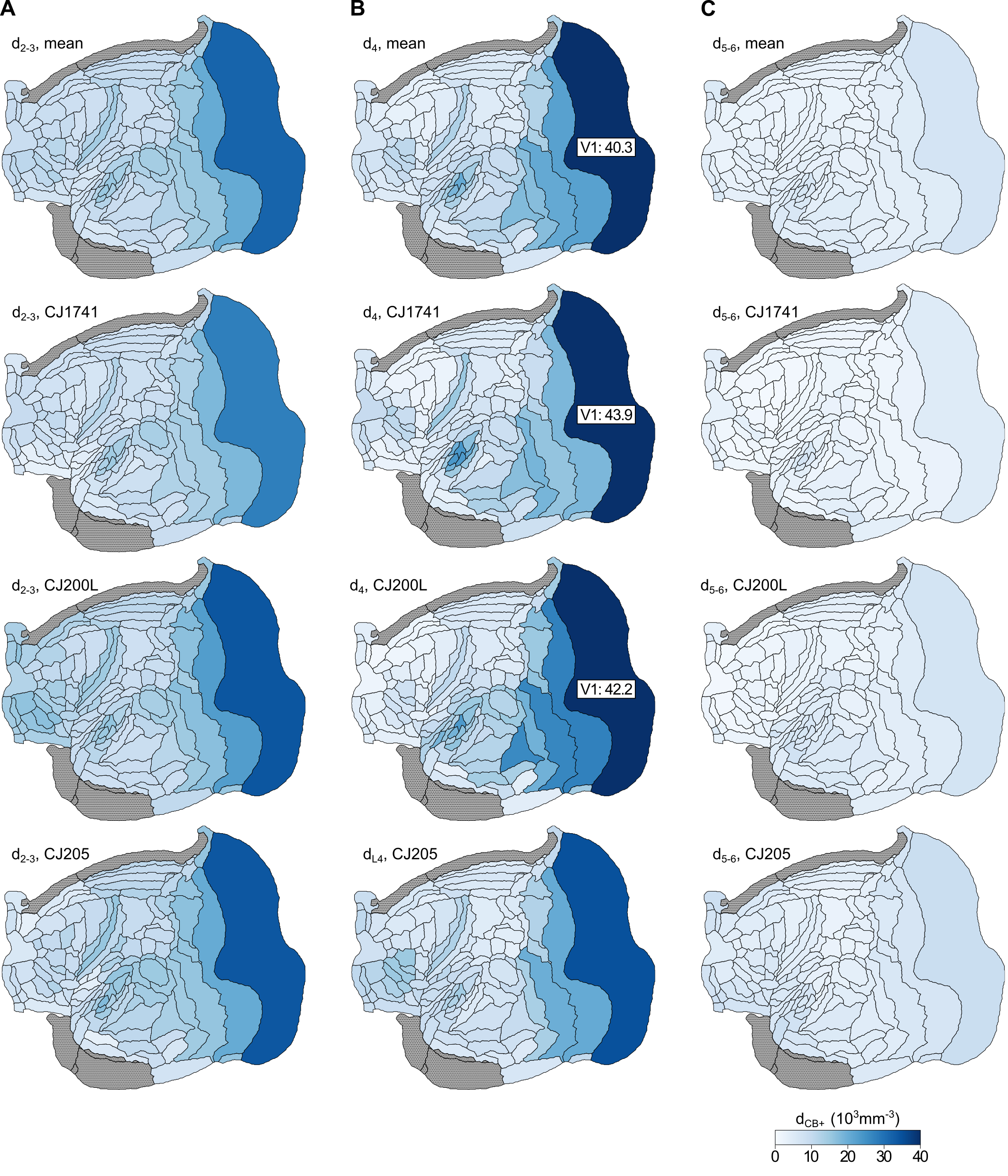

Supplement: S2 Fig — (A) Supragranular layers 2 and 3. (B) Layer 4. (C) Infragranular layers 5 and 6. Each column shows maps for the average of the three cases (top row) followed by the three individual marmosets. For each map, the scale (bottom right) indicates densities in neurons·mm-3. (TIF) [file pcbi.1012428.s002.tif]

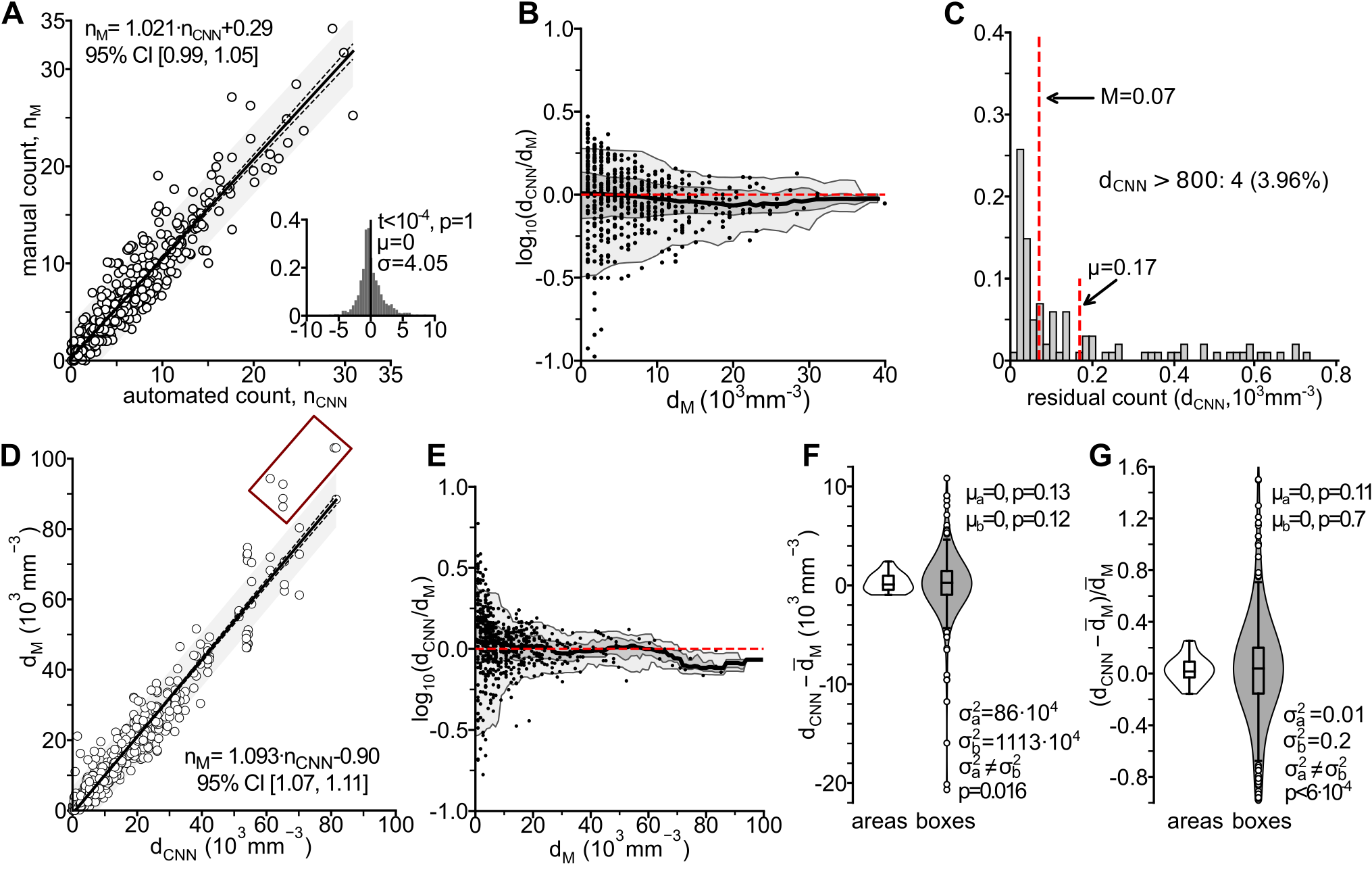

Supplement: S3 Fig — (A) The evaluation of the automatic cell counting performance using the validation dataset (603 counting boxes). The relation between the automated count (nCNN, abscissas) and the ground truth manual count (nM, ordinates) shows a linear relation between the two quantities. The residuals average to zero (two-sided t-test, t<10−4, p = 1, μ = 0). (B) Relative error of the U-Net CNN (dCNN) neuronal densities against those established by manual counting (dM). Black points represent results for the individual counting boxes. Order statistics (median: thick black line, the light gray bands: 5th and 95th centiles, dark gray: lower and upper quartiles) calculated locally within a 5·103mm-3 wide moving window. The red dashed line represents the agreement between the U-Net CNN and the manual results. The relative error decreases with increasing CB+ densities (the bands are wider for lower densities and narrower for higher ones). (C) Histogram of the residual densities (i.e. the density estimated by the U-Net CNN within image patches known to contain no neurons). Among the 2,216 empty image patches, 50% had a residual density less or equal to 70 mm-3, and the mean residual density amounted to 170 mm-3, which shows that the impact of the background on the U-Net CNN results is negligible. (D) Comparison of the CB+ densities estimated by the U-Net CNN against three expert neuroanatomists. Densities established by the U-Net CNN (dCNN, abscissas) against the count obtained manually (dM, ordinates) for each image patch in these areas (939 values in total) show a linear relation between the two quantities. (E) Relative error of the U-Net CNN (dCNN) neuronal densities against manual counting (dM) for the benchmark dataset. Black points represent results for the individual image patches. Order statistics (median: thick black line, light gray bands: 5th and 95th centiles, dark gray: lower and upper quartiles) calculated locally within a 5·103 mm-3 wide moving window. The dashed red line [file pcbi.1012428.s003.tif]

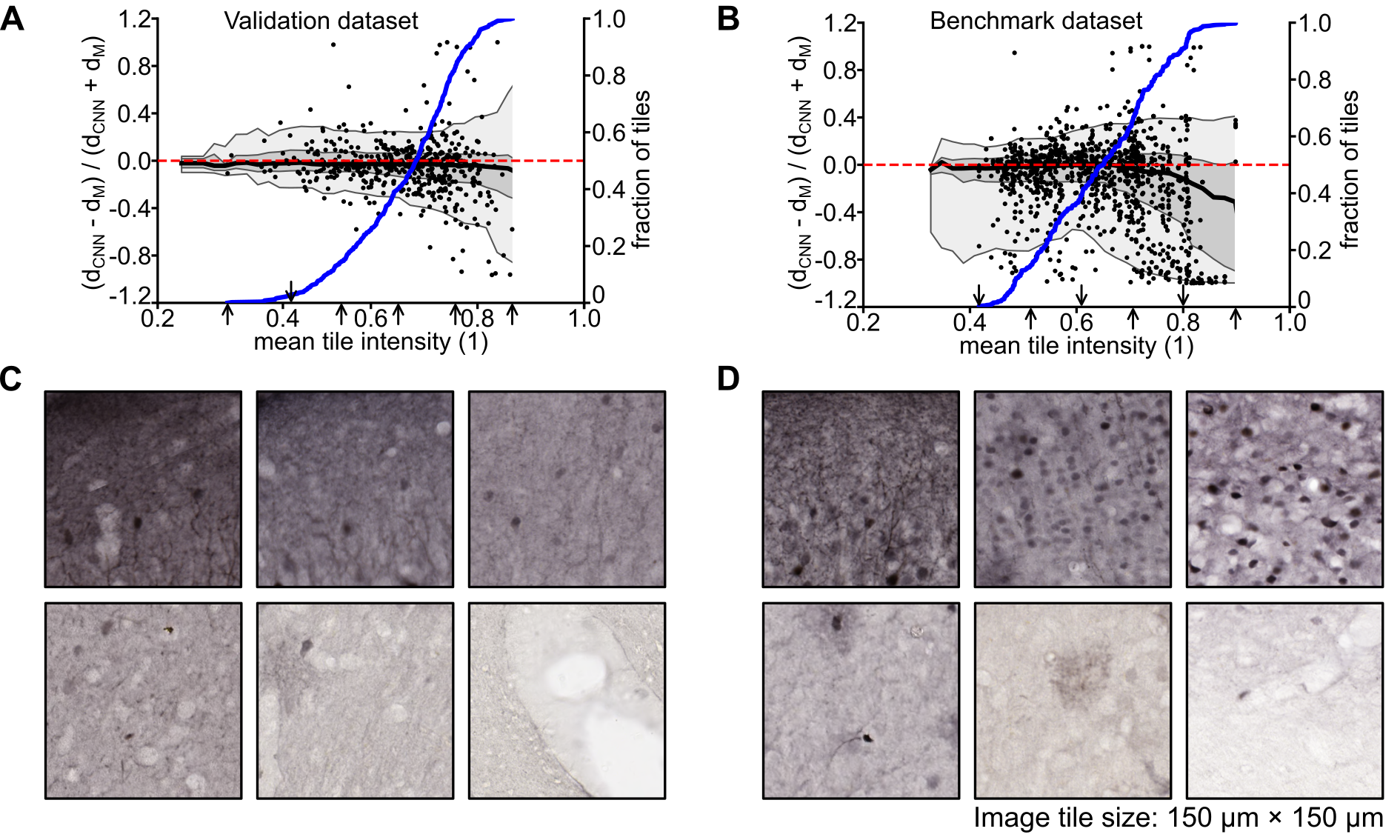

Supplement: S4 Fig — Relation between the mean tile intensity of the validation (panel A) or the benchmark dataset (panel B) and the relative error of the U-Net CNN (dCNN) neuronal densities against those established by manual counting (dM) presented as relative difference: (dCNN-dM) / (dM+dCNN). The mean tile intensity is based on all three color channels and then normalized to the 0–1 range. Black points represent results for the individual counting boxes. Order statistics (median: thick black line, the light gray bands: 5th and 95th centiles, dark gray: lower and upper quartiles) calculated locally within a 0.05 wide moving window. The red dashed line represents the agreement between the U-Net CNN and the manual results. The thick blue curve represents the fraction of tiles of equal or lower mean intensity (cumulative distribution). The up (↑) or down (↓) arrows on the abscissa axis represent the mean tile intensity values for which examples of image tiles are provided in panels C (for the validation dataset) and D (for the benchmark dataset). The relative error increases with mean tile intensity (the bands are wider for higher mean intensity and narrower for lower), however, it remains relatively stable up to the average image intensity of approximately 0.75, which corresponds to approximately 80% of image tiles for which the relative error could be described as independent from the mean image intensity. It has to be noted that the mean tile intensity of 0.75 and higher (gray level of 192 in 8-bit images) corresponds mainly to bright image tiles (see the bottom center and the bottom right examples in panels C and D) mainly from the bottom of the strips (e.g., deep layer VI or white matter). Such tiles typically have only a few cells which exaggerates the relative error due to the division by small numbers. Overall, these findings suggest that the U-Net model is similarly affected by staining variation as the manual plotting. (TIF) [file pcbi.1012428.s004.tif]

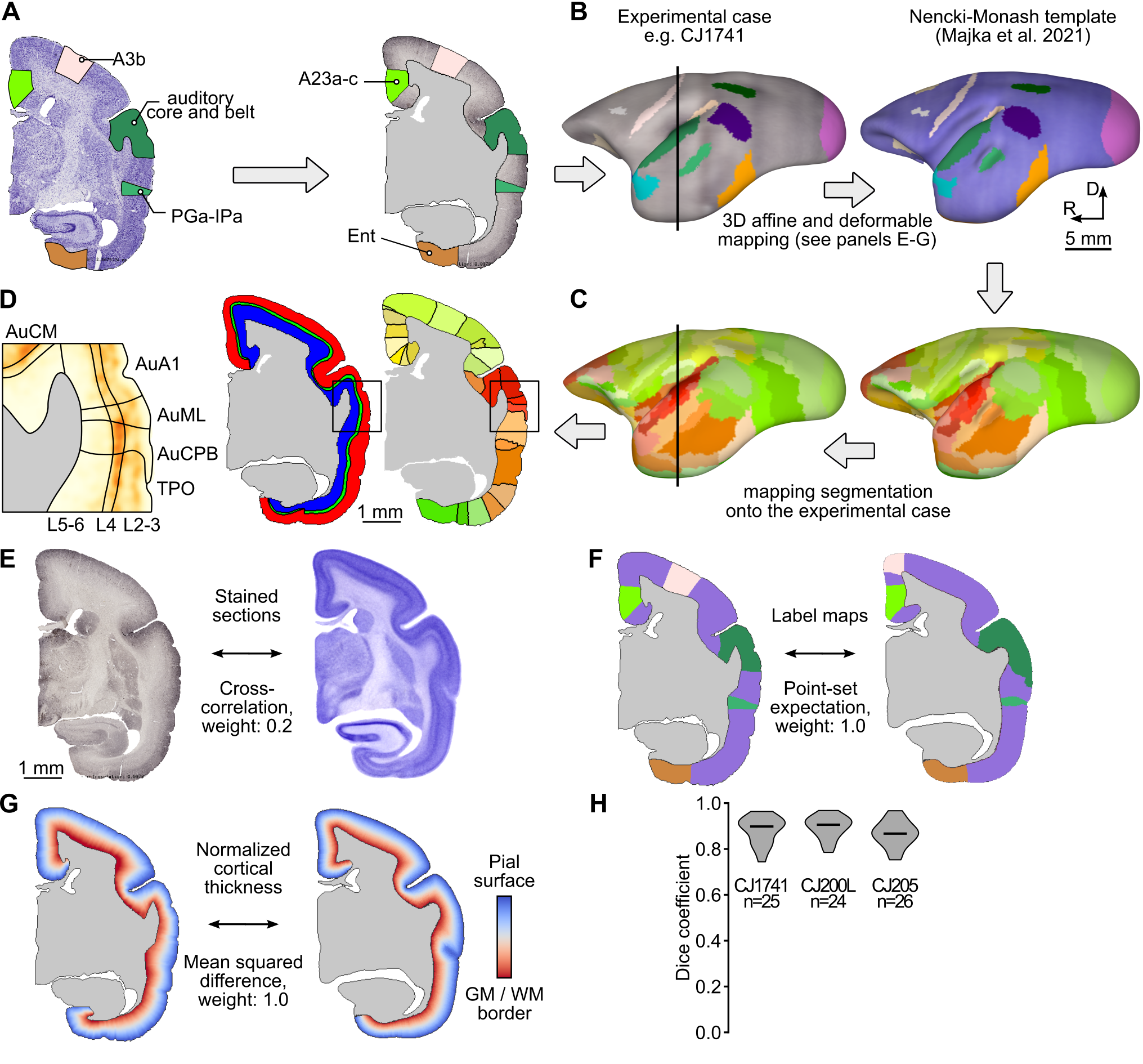

Supplement: S5 Fig — (A) Label maps outlined manually on Nissl sections (left) are transferred onto the adjacent CB-stained sections (right). (B) Registration of the experimental case to the reference template. A combined view of the CB-stained sections and the outlined label maps (left) in the experimental case, the Nissl-stained section, and the label maps in the reference template (right). (C) The computed spatial transformations are used to map the segmentation from the reference template (right) onto the experimental case (left). The black line indicates the coronal location of the section shown in panels A and D–G. (D) Example CB-stained coronal section segmented into cortical areas (right), manual segmentation of the same section into supragranular, granular, and infragranular layers (red, green and blue, respectively, middle). The combination of the areal and laminar segmentation allows for computing densities of CB+ neurons in individual areas across different layers (left). See S1 File for a full list of areas, color coding and abbreviations. (E–G) Pairs of images and respective metrics used to simultaneously drive the registration. (E) Cross-correlation (CC, [89]) between grayscale images of the stained sections, here shown in color for clarity. (F) Point-Set Expectation (PSE, [88]) metric forces corresponding label maps from the experimental case and the template to overlap. See S3 Table for a detailed list of label maps delineated for each case. (G) Normalized cortical thickness maps calculated using a 2-surface Laplacian-based approach [32,90]. Zero (blue color) corresponds to the pial surface, while one (red) to the border between the gray and the white matter. (H) Registration accuracy expressed with the Dice coefficient between individual label maps. The violin plots represent distributions of the overlap values between pairs of corresponding maps. The thick black lines represent the median values of the Dice coefficient for all label maps in each case. See S3 Table for [file pcbi.1012428.s005.tif]

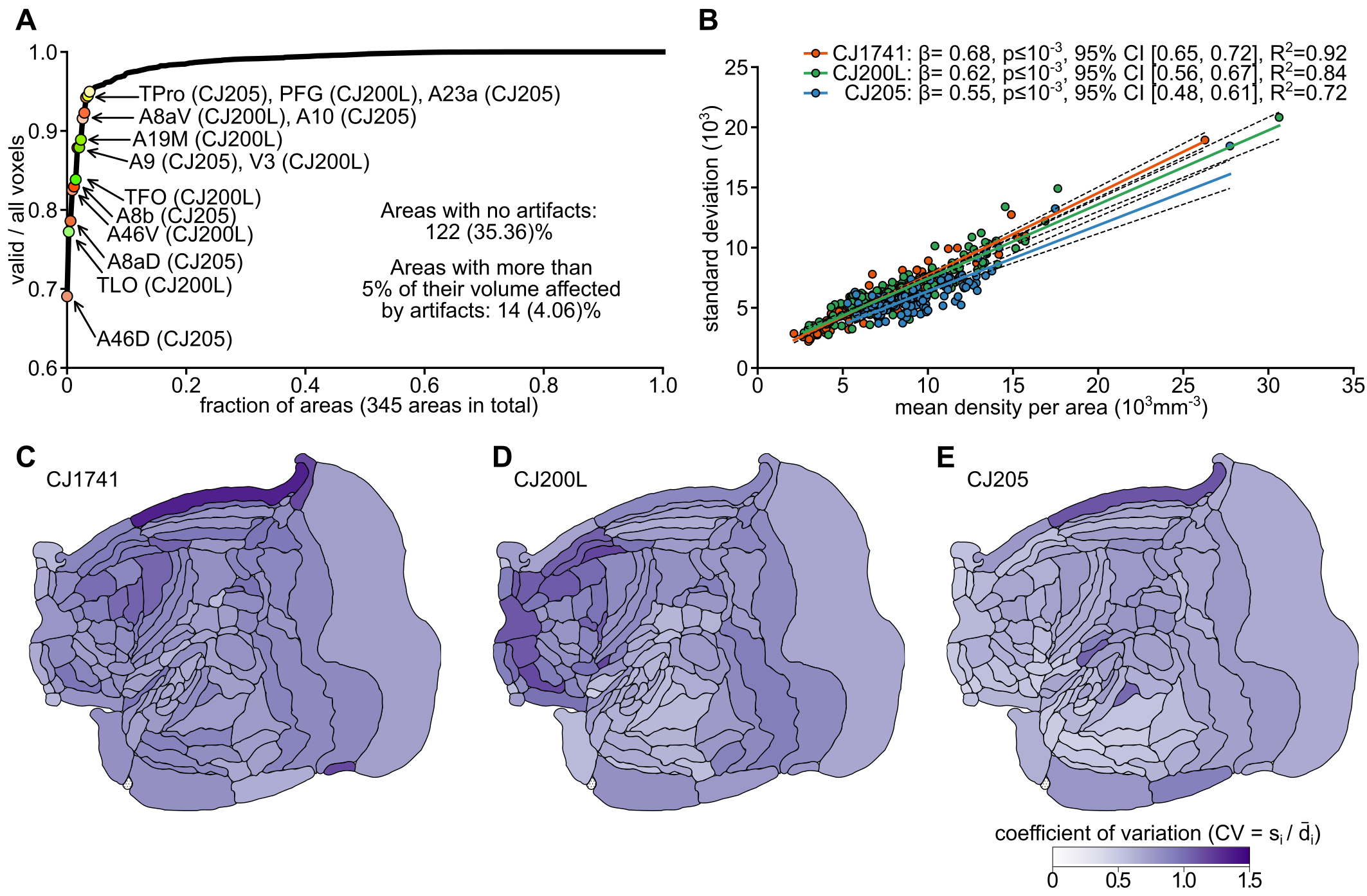

Supplement: S6 Fig — A) Relation between the ratio of voxels annotated as artifacts against the total number of voxels per area in all three brain hemispheres. For clarity, data for all 345 areas are plotted with a thick black line. Areas with more than 5% of their volume affected by artifacts are displayed with color-coded points and annotated. Out of all areas, over 35% did not suffer from a single artifactual voxel, while only slightly over 4% of areas (14 out of 345) had more than 5% of their volume annotated as artifacts. In other words, almost 96% of areas had fewer than 5% of voxels excluded from analysis. B) Linear relation between an area’s mean CB+ neuronal density and its standard deviation. The points represent the mean density per area in each case (red–CJ1741, green–CJ200L, blue–CJ205), the color lines represent the best linear fit for each case, and the dashed black lines indicate the 95% confidence interval for the fit. The results are consistent among the three cases and indicate a strong dependence of the dispersion on the mean value of a sample (i.e. heteroskedasticity). This implies caution when using the standard deviation to interpret the variance of densities. C-E) Maps of the coefficient of variation (ratio of the standard deviation to the mean, CV = si / d¯i) for all areas in three animals reveal no clear patterns of the degree of variation. (TIF) [file pcbi.1012428.s006.tif]
