# Supplementary material for: Distribution of calbindin-positive neurons across areas and layers of the marmoset cerebral cortex
Source: PLoS Comput Biol. 2024 Sep 23;20(9):e1012428. doi: 10.1371/journal.pcbi.1012428 (PMC11495585; doi:10.1371/journal.pcbi.1012428)
Supplement: S1 File — (PDF) [file pcbi.1012428.s010.pdf]

Abbreviations of the names of cortical areas

Group	Abbreviation	Name
DLP	A10	area 10 of cortex
	A9	area 9 of cortex
	A46V	area 46 of cortex ventral part
	A46D	area 46 of cortex dorsal part
	A8aD	area 8a of cortex dorsal part
	A8b	area 8b of cortex
	A8aV	area 8a of cortex ventral part
VLP	A47L	area 47 (old 12) of cortex lateral part
	A47M	area 47 (old 12) of cortex medial part
	A45	area 45 of cortex
	A47O	area 47 (old 12) of cortex orbital part
	ProM	proisocortical motor region (precentral opercular cortex)
OFC	A11	area 11 of cortex
	A13b	area 13b of cortex
	A13a	area 13a of cortex
	A13L	area 13 of cortex lateral part
	A13M	area 13 of cortex medial part
	OPAl	orbital periallocortex
	OPro	orbital proisocortex
	Gu	gustatory cortex
MPC	A32	area 32 of cortex
	A32V	area 32 of cortex ventral part
	A14R	area 14 of cortex rostral part
	A14C	area 14 of cortex caudal part
	A25	area 25 of cortex
	A24a	area 24a of cortex
	A24b	area 24b of cortex
	A24c	area 24c of cortex
	A24d	area 24d of cortex
MPM	A6DR	area 6 of cortex dorsorostral part
	A6Vb	area 6 of cortex ventral part b
	A6Va	area 6 of cortex ventral part a
	A8C	area 8 of cortex caudal part
	A6M	area 6 of cortex medial (supplementary motor) part
	A6DC	area 6 of cortex dorsocaudal part
	A4c	area 4 of cortex part c (primary motor)
	A4ab	area 4 of cortex parts a and b (primary motor)
INS	PaIM	parainsular cortex medial part
	AI	agranular insular cortex
	PaIL	parainsular cortex lateral part
	DI	dysgranular insular cortex
	GI	granular insular cortex
	IPro	insular proisocortex
	TPro	temporal proisocortex
SSC	S2PR	secondary somatosensory cortex parietal rostral area
	A3a	area 3a of cortex (somatosensory)
	S2PV	secondary somatosensory cortex parietal ventral area
	A3b	area 3b of cortex (somatosensory)
	S2I	secondary somatosensory cortex internal part
	S2E	secondary somatosensory cortex external part
	A1-2	areas 1 and 2 of cortex
AUD	AuRTL	auditory cortex rostrotemporal lateral area
	AuRT	auditory cortex rostrotemporal
	AuRPB	auditory cortex rostral parabelt
	AuRTM	auditory cortex rostrotemporal medial area
	AuR	auditory cortex rostral area
	AuRM	auditory cortex rostromedial area
	AuAL	auditory cortex anterolateral area
	AuA1	auditory cortex primary area
	AuCM	auditory cortex caudomedial area
	AuCPB	auditory cortex caudal parabelt area
	AuML	auditory cortex middle lateral area
	AuCL	auditory cortex caudolateral area
LIT	TPPro	temporopolar proisocortex
	STR	superior temporal rostral area (cortex)
	TE1	temporal area TE1 (inferior temporal cortex)
	TPO	temporo-parieto-occipital association area (superior temporal polysensory cortex)
	ReI	retroinsular area (cortex)
	TE2	temporal area TE2 (inferior temporal cortex)
	PGa-IPa	area PGa and IPa (fundus of superior temporal ventral area)
	TPt	temporoparietal transitional area
	TE3	temporal area TE3 (inferior temporal cortex)
	TEO	temporal area TE occipital part
VTC	Pir	piriform cortex
	Ent	entorhinal cortex
	A36	area 36 of cortex
	A35	area 35 of cortex
	TF	temporal area TF
	TL	temporal area TL
	TH	temporal area TH
	TLO	temporal area TL occipital part
	TFO	temporal area TF occipital part
PCR	A23c	area 23c of cortex
	A23a	area 23a of cortex
	A29d	area 29d of cortex
	A30	area 30 of cortex
	A23b	area 23b of cortex
	A29a-c	area 29a-c of cortex
	A23V	area 23 of cortex ventral part
	ProSt	prostriate area
PPC	PF	parietal area PF (cortex)
	PE	parietal area PE
	PFG	parietal area PFG (cortex)
	A31	area 31 of cortex
	AIP	anterior intraparietal area of cortex
	PG	parietal area PG
	PEC	parietal area PE caudal part
	VIP	ventral intraparietal area of cortex
	LIP	lateral intraparietal area of cortex
	PGM	parietal area PG medial part (cortex)
	V6A	visual area 6A (posterior parietal medial area)
	OPt	occipito-parietal transitional area of cortex
	MIP	medial intraparietal area of cortex
VIS	MST	medial superior temporal area of cortex
	FST	fundus of superior temporal sulcus area of cortex
	V5	visual area 5 (middle temporal area)
	V4T	visual area 4 transitional part (middle temporal crescent)
	A19M	area 19 of cortex medial part
	V3A	visual area 3A (dorsoanterior area)
	V4	visual area 4 (ventrolatereral anterior area)
	V6	visual area 6 (dorsomedial area)
	A19DI	area 19 of cortex dorsointermediate part
	V3	visual area 3 (ventrolateral posterior area)
	V2	visual area 2
	V1	primary visual cortex

Abbreviations of individual areas follow the nomenclature proposed by Paxinos G, Watson C, Petrides M, Rosa M, Tokuno H. The marmoset brain in stereotaxic coordinates. Elsevier Academic Press; 2012.

Abbreviations of the groups of cortical areas

Abbreviation	Name
DLP	Dorsolateral prefrontal cortex
VLP	Ventrolateral prefrontal cortex
OFC	Orbitofrontal cortex
MPC	Medial prefrontal cortex
MPM	Motor and premotor cortex
INS	Insula and rostral lateral sulcus
SSC	Somatosensory cortex cortex
AUD	Auditory cortex
LIT	Lateral and inferior temporal cortex
VTC	Ventral temporal cortex
PCR	Posterior cingulate, medial and retrosplenial cortex
PPC	Posterior parietal cortex
VIS	Visual cortex

Adapted from Reser DH, Burman KJ, Yu H-H, Chaplin TA, Richardson KE, Worthy KH, Rosa MGP. Contrasting patterns of cortical input to architectural subdivisions of the area 8 complex: a retrograde tracing study in marmoset monkeys. Cereb Cortex. 2013;23: 1901-22. doi: 10.1093/cercor/bhs177.

Location of marmoset cortical areas on two-dimensional flatmap

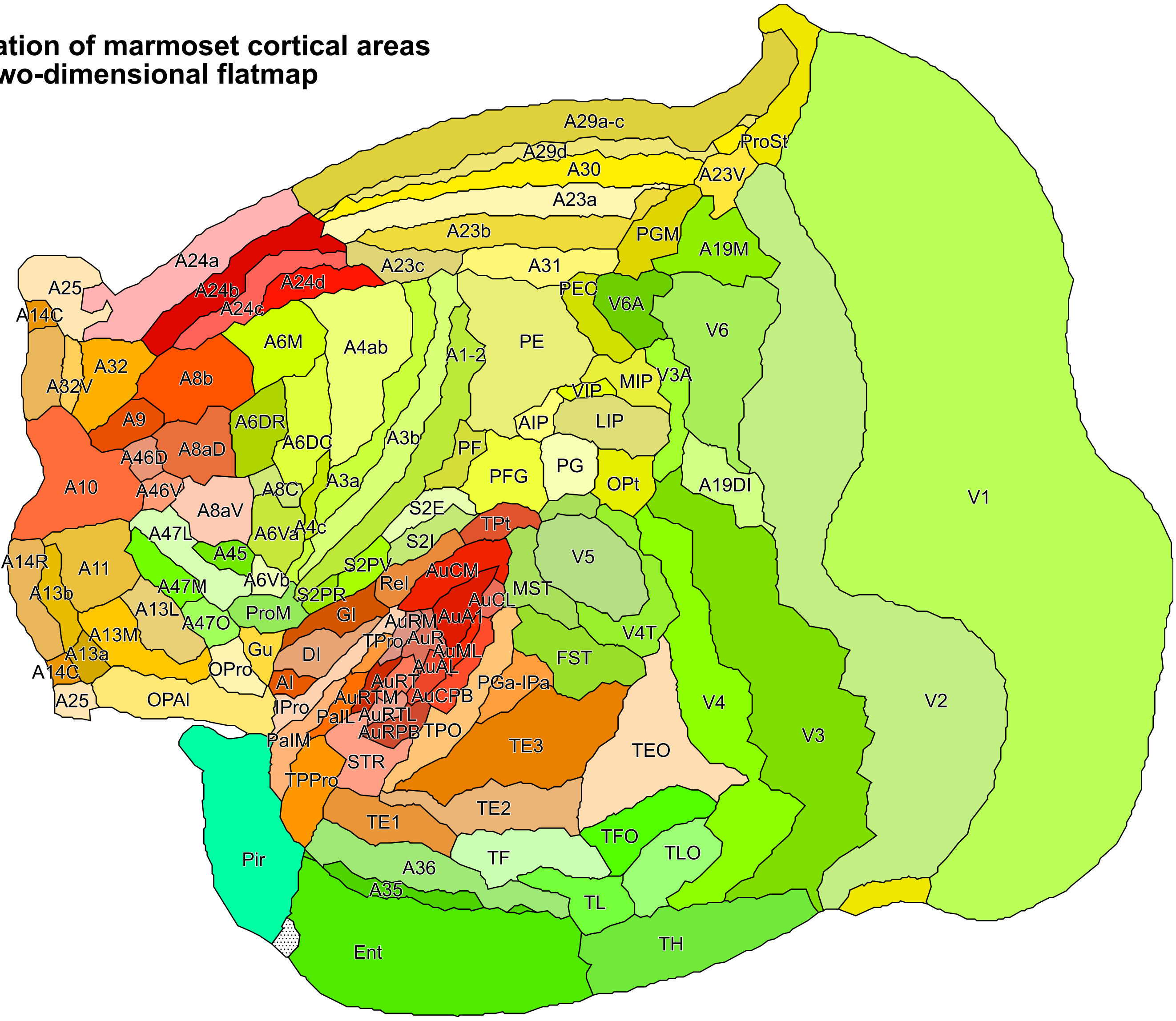

Groups of cortical areas

Related to figs. 3, 4, 6, and S5.

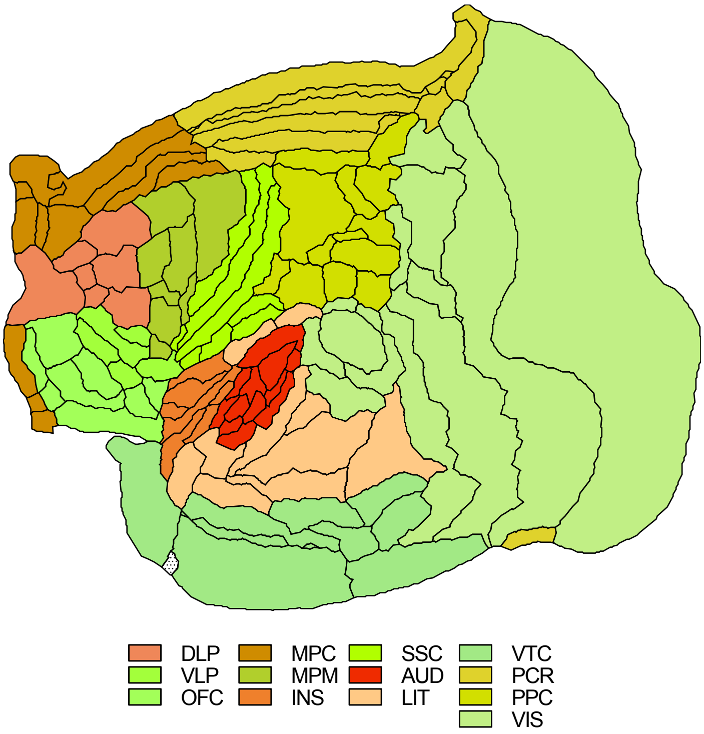

Cytoarchitectural categories of lamination

Related to figs. 3 and 7.

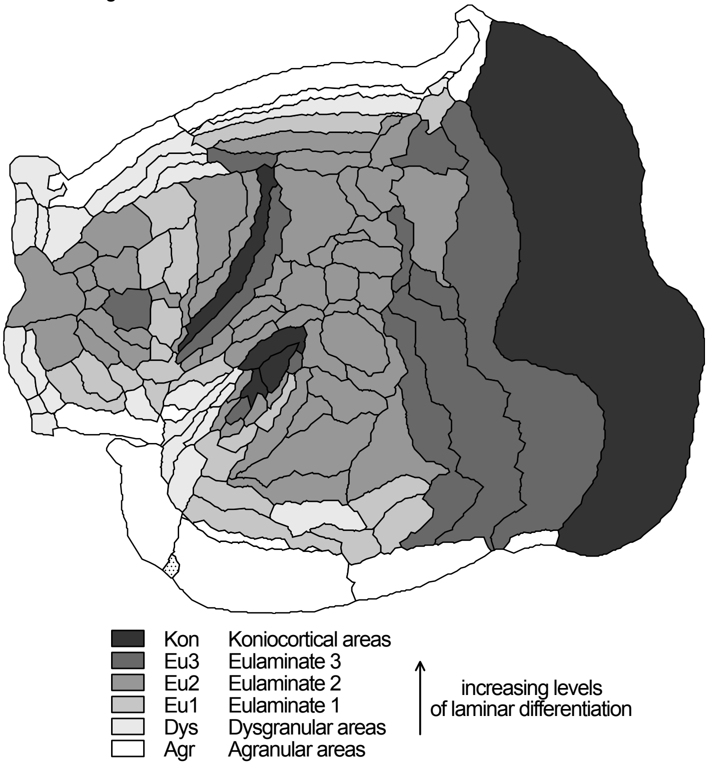

Adapted from John YJ, Zikopoulos B, García-Cabezas MÁ, Barbas H. The cortical spectrum: A robust structural continuum in primate cerebral cortex revealed by histological staining and magnetic resonance imaging. Front Neuroanat. 2022;16: 897237. doi: 10.3389/fnana.2022.897237.
