# Supplementary material for: Distribution of calbindin-positive neurons across areas and layers of the marmoset cerebral cortex
Source: PLoS Comput Biol. 2024 Sep 23;20(9):e1012428. doi: 10.1371/journal.pcbi.1012428 (PMC11495585; doi:10.1371/journal.pcbi.1012428)
Supplement: Attachment — Submitted filename: reply_to_reviewers.pdf [file pcbi.1012428.s011.pdf]

Dr. Lyle Graham
Section Editor
PLOS Computational Biology

9 July 2024

**Re: Decision from PLOS Computational Biology (PCOMPBIOL-D-24-00511)**

Dear Dr. Graham

Thank you for the correspondence dated 22 May, informing us of the outcomes of the first round of reviews of our manuscript titled "Distribution of calbindin-positive neurons across areas and layers of the marmoset cerebral cortex."

Here, we take the opportunity to submit a revised version, which, as requested, considers the points raised by the Reviewers. The most prominent changes were in the form of new figures and tables related to the newly performed analyses:

1. Evaluation of the automated cell counting method against staining intensity (Supporting Figure S4 and Supporting Table S2).
2. Additional insights into data quality and individual variation in the density of $CB^+$ neurons (Supporting Figure S6).
3. Extension of the existing Supporting Figure S5 and the "Methods" section with details on the registration accuracy and list of delineated label maps in each hemisphere (Supporting Table S3).

In addition, we have significantly extended the discussion, particularly the "Limitations of the study" section. Further, the entire manuscript has been carefully revised, including several new minor passages and amendments to the figures. As per your suggestion, the changes are indicated in the "tracked changes" version of the manuscript.

We hope that these revisions meet the requirements and expectations.

Yours truly,

Nafiseh Atapour
Marcello Rosa
Piotr Majka

**Nencki Institute of Experimental Biology,** Polish Academy of Sciences
3 Pasteur St., 02-093 Warsaw, Poland
phone: +48 22 58 92 207, email: dyrekcja@nencki.edu.pl
**www.nencki.edu.pl**

## REPLY TO REVIEWERS

*In the text below, Q refers to the reviewers' original questions, and A to our answers, including the actions taken.*

***Reviewer #1***

*The present study examines the 3D distribution of calbindin-positive (CB+) neurons across the entire marmoset cortex. It uses immunohistochemistry for imaging the CB+ neurons, and proposes a workflow consisting of automatic cell density estimation and 3D reconstruction of histological sections to extract the density distribution and compute quantitative maps organized by cytoarchitectonic structures. The approach offers valuable novel insights in the cytoarchitecture of the primate brain, revealing distinct patterns of CB+ neuron distribution across various cortical areas and layers, as well as the relationship to general neuronal densities. The reported results should be of significant interest to the community.*

*The experiments are based on serial sections of one hemisphere of each of three young adult marmoset brains, where immunohistochemistry stains are applied to each 5th section. Of the remaining sections, adjacent ones were stained for NeuN, myelin and cytochrom oxidase to identify areas and layers. Quantification of cell densities is based on a conventional U-Net model which is trained to predict cell numbers of image patches in the form of sparse Gaussian kernel density maps. The network is trained from expert annotations in sample strips for each studied brain region extracted from one of the cases. Validation of the trained model is done against expert annotations in a selection of brain areas in all three brains.*

*The paper is well written and organized. The experimental results are comprehensive, convincing, and well presented. The data and machine learning model are provided alongside the paper, including the labelled training dataset, which is much appreciated to reproduce the results.*

We thank the reviewer for her / his insightful comments and suggestions and for appreciating our work.

*However, I identified a couple of minor issues that should be taken into account to improve the manuscript:*

*Q1: While the paper claims at several places (e.g. introduction, beginning of results section, top of page 15) to study all lamina of the cortex, the actually reported results refer to groups of lamina (2/3, 4, 5/6). This should be properly reflected everywhere in the text.*

A: We have edited several sentences in response to this comment to make our statements more precise. We have also included an additional paragraph in the *Limitations of the study* section to acknowledge this particular drawback.

**Nencki Institute of Experimental Biology,** Polish Academy of Sciences
3 Pasteur St., 02-093 Warsaw, Poland
phone: +48 22 58 92 207, email: dyrekcja@nencki.edu.pl
**www.nencki.edu.pl**

nencki institute
of experimental biology

*Q2: I was wondering why there are no standard deviations displayed for density estimates, such as in Fig. 4 and Fig. 6. Isn't it so that densities are predicted for sets of small patches and averaged per region/region group? If yes, I suggest displaying them in these figures. This would also help to interpret the variance of densities across the three cases better, which is quite significant in some cases (e.g. OFC).*

A: We discussed this matter before the initial submission and, following the motivation presented below, decided against displaying this information on the plots.

Indeed, the average density for an area or layer is computed by averaging the density of all voxels belonging to the particular area or layer. However, the distribution of the $CB^+$ cells within an area (or even a group of layers) is not uniform (see, for instance, Figure 2F or Supporting Figure S5D), which suggests that the standard deviation might not be an appropriate measure to describe the density variation as it would oversimplify the complex cytoarchitectural patterns in an area or group of layers.

More importantly, perhaps, we identified a linear relation between the mean density in an area and its standard deviation (see newly added Supporting Figure S6B). This indicates that the standard deviation would not be informative as it strongly correlates with the average density. The relation where the dispersion depends on the sample's mean is called heteroscedasticity and is a purely statistical phenomenon. Therefore, presenting the standard deviation could be misleading by suggesting a genuine physiological phenomenon while dealing with an exclusively statistical effect.

Please note that while we decided not to display the standard deviations (or any other measures of dispersion), the calculated values are available in the newly added Supporting Table 2.

For completeness, we also calculated the coefficient of variation (the ratio of the standard deviation to the mean density, $CV = \overline{d}_i / s_i$) for each area (Supporting Figure S6 C–E). This coefficient, while somewhat more robust than the standard deviation, also did not reveal any consistent patterns of variability between the three animals, highlighting the complexity of this phenomenon.

In summary, in response to this remark, we have 1) added Supporting Figure S6 B–E and 2) slightly altered the *Mapping cell distributions across the entire cortex* to highlight the necessity of further studies on the variability.

*Q3: Somewhat related, I found it rather hard to compare the density values in Fig 4 between the three cases, due to the way the barplots are organized into split columns. It seems to me that if the bar plots were transposed, one could have one row per case with corresponding areas vertically aligned, then visually much closer to each other and better to read. Did you try such layout?*

A: We rearranged the figure according to the reviewer's specifications. We agree that the new layout better illustrates the relative differences between $CB^+$ densities in each case and how consistent these relations are.

*Q4: While the registration workflow has been published earlier by Majka et al., I find the description and evaluation of the approach a bit short in the paper. In particular, it could be mentioned between which images the correlation metric is evaluated. More importantly, although I have no reason to assume insufficient registration quality, it would be helpful to see some quantitative assessment of the accuracy. On p. 27 it is mentioned that adding the label maps to guide the registration improves the accuracy, but I did not find any explanation how this was measured and how significant the effect was.*

A: Given that the registration workflow has been comprehensively described in our previous publications, we intended to limit the details to avoid inflating the current article with repetitive information. However, pursuing the Reviewer's request, we took the following actions to address this remark:

1. Provided more details of the registration procedure, including a clear indication of which pairs of images and similarity metrics were used.
2. Updated Figure S5 (formerly Figure S4) with examples of image pairs used for the registration.
3. Compiled the list of label maps delineated in individual brain hemispheres and assessed the coregistration accuracy by computing the Dice coefficient for each label map (newly added Supporting Table 3 and Figure S5G).

Label maps are a powerful but relatively rare approach. They were actually temporarily removed from ANTS but, fortunately, reimplemented in the recent versions. Label maps allow for additional, expert-based spatial constraints which significantly reduces the likelihood of falling into a local minimum of the similarity metric, which typically translates into gross misregistration such as mismatched sulci. In our previous study (Majka et al., 2021), we found that the use of label maps improved segmentation accuracy in all examined areas, especially for smaller ones with complex morphology or those located deep in sulci (Figure S5 in https://ars.els-cdn.com/content/image/1-s2.0-S1053811920311101-mmc1.pdf). Although we did not conduct these tests for this article due to their time-consuming nature, we anticipate a similar positive impact of label maps on registration accuracy.

Q5: *I did not find an explanation about which hemispheres were used in each case, and why it was decided to only analyze one hemisphere per brain. This should be briefly explained.*

A: A sentence was added to the first paragraph of the *Experimental model and subject details* section to explain that we used intact hemispheres from animals that also had tracer injections in the other hemisphere.

*Q6: It is mentioned that tissue distortions were masked and excluded from evaluations. It would be helpful to report the average amount of corrupt tissue, and especially valuable to provide these masks together with the data.*

A: We fully agree with the Reviewer. The masks were provided in the initial submission as supporting NIFTII files compatible with the rest of the dataset (density maps and segmentations into areas and layers). These binary masks (named `CJ1741_mask.nii.gz`,

`CJ200L_mask.nii.gz`, and `CJ205_mask.nii.gz`) indicate which voxels are to be excluded from the analyses (voxels of value 0) due to tissue distortions (e.g., tears or folds) or staining artifacts.

Expanding on the Reviewer's request, we measured, for each area, the ratio of voxels annotated as artifacts to the total number of voxels in the area. The results are available as Supporting Figure S6A. The analysis highlights that over 35% of areas in all cases did not suffer from a single artifactual voxel. In comparison, only slightly over 4% of areas (14 out of 345) had more than 5% of their volume annotated as artifacts. In other words, almost 96% of areas have less than 5% of voxels excluded from analysis. Upon inspecting the 14 areas with the highest percentage of corrupted tissue, we found no clear situation where the artifacts would cause a qualitative difference in density estimate in a particular case vs. the other two (i.e., when the estimate in a particular area in a specific case was clearly against the trend established by the two other cases). We provide the source data behind the plot in Supporting Figure S6A in the newly added Supporting Table S2. Additionally, we updated Fig. 2G to show the exclusion mask on top of the density map.

*Q7: The discussion of alternative cell detection methods on page 20 is a bit outdated and selective. I would suggest to briefly mention the current state of the art represented by the recent NeuRIPs challenge (Ma et al., Nat Meth 2024, https://doi.org/10.1038/s41592-024-02233-6). Of course I do not expect to repeat any experiments with other instance segmentation models, since the authors do not claim to use the best available method and explicitly encourage to try other models using the published training data.*

A: We amended this part of the discussion to include examples of state-of-the-art transformer-based segmentation methods and clarify their advances over CNN-based approaches, as presented in the suggested review paper.

*Q8: The excluded brain areas discussed on p. 27 (bottom) should be reported with the limitations of the approach.*

A: This limitation is now mentioned at the end of a new paragraph added at the end of the *Limitations of the study* section.

*Q9: The introduction motivates that there are comprehensive existing resources on distributions of cortical neurons in the marmoset brain. I would have expected that at least some of these published measures are mentioned in the discussion later on.*

A: We have edited the last paragraph of the *Introduction* to address this comment.

*Some additional things I came across:*

*Q10: In the caption of Fig. 2, the sentence starting with "Upon training..." seems to have some misplaced commas.*

A: Thank you for pointing out the mistake. It has been corrected.

*Q11: I was wondering if the coloring of the brain area groupings (e.g. Fig. 3A) coul be used in the coloringn of the flat maps (e.g. Fig3D) as well. This would make it easier to interpret the figure, especially since the group names or not shown in the flat map either.*

After several attempts to introduce color-coding to the flatmaps and other figures, we concluded that this additional layer of information reduces the figure's readability rather than improves it. Instead, we created a reference card that summarizes the names, abbreviations, color codes, and flatmap locations of the individual cortical areas and groups of areas. It is available as a newly added Supporting File S1, and we hope it will provide the context for easier interpretation of the figures. We also amended the legends of relevant figures to refer to the reference card.

*Q12: I suggest to annotate figures consistently with the case IDs where applicable to improve the connection between them (e.g. in Figure 4 they are not given).*

A: We believe all figures were annotated with case identifiers as the plots are generated by scripts. In this particular figure, the case identifier was placed at the bottom of the second column for each hemisphere (below the density estimates for the visual areas, just under the density scale). We agree this was not the most fortunate location. Since this figure has been reworked, in the revised version, we placed the case identifier in a much more prominent location – next to the panel name (A, B, C). We have also revised other relevant figures to ensure the case identifiers are easily noticeable.

***Reviewer #2***

*The manuscript of Majka et al. describes their work mapping the distribution of calbindin-positive neurones in the marmoset cerebral cortex. The result is very interesting, as it provides the first extensive map of a neuronal type in a widely used non-human primate brain. Even more interesting, their rigorous and well thought methodology should provide a template for further studies of other markers and other species. This methodology succeeds in combining input from experts (manual segmentation and annotation) with sophisticated computational neuroanatomy analyses. It is this combination which enables them to scale the focused work of experts to the whole cerebral cortex, providing analyses at the level of cytoarchitectonic areas as well as cortical layers. It is highly commendable that their data (including the annotations used for training) and their code are made openly accessible and open source.*

*The manuscript is well written, very clear; the illustrations are also clear and informative; and information about statistical analyses seems adequate to assess the validity of their results (interested readers will also be able to download data and code if they want to perform additional validation).*

We would like to thank the Reviewer for the positive reception of our work and constructive comments. We hope that our revisions address the Reviewer's remarks.

*Q1: I have only one, minor, question: despite the careful approach to 3D reconstruction and estimation of neuronal densities, the map in figure 2G shows vertical coronal bands which suggest an artefact in density estimation due to the slicing plane. Could the authors discuss a bit the origin of this artefact?*

A: After examining this particular figure, along with the $CB^+$ density map for this case (CJ1741) and density maps for other cases, we have identified several reasons behind the presence of the vertical coronal bands. The main issue stems from the unavoidable variability in staining intensity despite carefully executed experimental protocols for immunohistochemical staining. Other reasons are related to the visualization itself (e.g. sampling the density maps with a smoothed model of the midthickness surface or selecting a color map that might exaggerate the perception of the vertical bands) and do not address the core issue.

As outlined in the methods section, we identified and excluded parts of the cortical tissue that showed severe staining artifacts such as the absence of labeled neurons despite the presence of cells in corresponding locations on adjacent sections or tissue corruption. These excluded areas were not originally depicted in Fig. 2G, which left some parts of the cortex that should have been excluded from the map exposed. We have updated Fig. 2G to show the exclusion mask superimposed on the density map.

*Q2: Would it be possible to quantify it or compensate it? Low frequency filtering could be a possibility, however, it may blur important information especially when rapid changes in density occur.*

A: The primary means we took to mitigate the negative effects of staining intensity variation was to make the model resilient to these artifacts. This was achieved by establishing a diverse training

dataset (e.g. Fig. 2A) and applying extensive image augmentation (in this context, primarily gamma and contrast variations to simulate the variability of the staining intensity) during the training.

In response to the reviewer's comment, we have compared the accuracy of the U-Net CNN and manual density estimates relative to the mean intensity of an image tile. The results are available as the newly added Supporting Figure S4 and show a good agreement between the U-Net CNN estimates and the manual counts for a broad range of image intensities and the vast majority of the data points. However, discrepancies are observed for bright image tiles with only a few cells. Please refer to the Supporting Figure S4 description for a detailed analysis. Overall, these findings suggest that the U-Net model is similarly affected by staining variation as the manual plotting.

We release the density maps in a spatially oriented form (NIfTI files) with the intention that other researchers could explore this dataset in the contexts not addressed in this study. Therefore, it is essential to preserve the original level of granularity by avoiding extensive postprocessing. Depending on the application, the dataset can be processed (including low-frequency filtering) to fit a particular analysis.

The striping artifacts do not affect the analyses in this article, which are based on average densities for combinations of areas and groups of layers. Those comprise voxels from several coronal sections, which mitigates the potential negative impact of the staining intensity variation between consecutive coronal sections.

In response to this remark, we took the following actions:

1. Updating Fig. 2G to present the exclusion mask superimposed on the density map.
2. Adding Supporting Figure S4 to illustrate how the model's performance depends on the image intensity.
3. Extending the *Limitations of the study* section with a paragraph concerning striping artifacts.

*Q3: Would it be imaginable to combine maps estimated from coronal sections with axial or sagittal maps?*

We understand that the Reviewer's question concerns the possibility of visualizing the density maps in planes other than coronal, including oblique cuts. As mentioned above, we release the density maps in NIfTI format, making it convenient to explore the dataset in spatially oriented contexts, particularly to visualize in off-coronal planes.
